# Asynchronous multi-decadal time-scale series of biotic and abiotic responses to precipitation during the last 1300 years

**DOI:** 10.1038/s41598-020-74994-x

**Published:** 2020-10-20

**Authors:** Sangheon Yi, Chang-Pyo Jun, Kyoung-nam Jo, Hoil Lee, Min-Seok Kim, Sang Deuk Lee, Xianyong Cao, Jaesoo Lim

**Affiliations:** 1 Korea Institute of Geoscience and Mineral Resources, Daejeon, 34132 Korea; 2 Korea University of Science and Technology (UST), Daejeon, 34113 Korea; 3 Kangwon National University, Chuncheon, 24341 Korea; 4 Nakdonggang National Institute of Biological Resources, Sangju, 37242 Korea; 5 Key Laboratory of Alpine Ecolgoy, Institute of Tibetan Plateau Research, Beijing, China; 6 CAS Center for Excellence in Tibetan Plateau Earth Sciences, Chinese Academy of Sciences (CAS), Beijing, 100101 China

**Keywords:** Climate sciences, Ecology

## Abstract

East Asian summer monsoon (EASM)-driven rapid hydroclimatic variation is a crucial factor with major socioeconomic impacts. Nevertheless, decadal- to centennial-scale EASM variability over the last two millennia is still poorly understood. Pollen-based quantitative annual precipitation (PqPann) and annual precipitation reconstructed by artificial neural networks (ANNs) for the period 650–1940 CE were reconstructed from a paleo-reservoir in South Korea. ANNs reconstruction was performed to compensate for a hiatus section. On a decadal timescale, 10 high-precipitation periods were identified, and PqPann and ANNs reconstructions were comparable to local instrumental rainfall and historic drought records. Biotic lags to rapid climatic changes ranging from 25 to 100 years were recognized by asynchronous pollen and speleothem responses to precipitation. We suggest that PqPann-based decadal- to centennial-scale climatic change reconstruction should take biotic lags into account, although the lags can be ignored on the millennial scale. The position of the EASM rainband influenced rainfall magnitude.

## Introduction

Abrupt climatic changes, including extreme events, accompanying global warming over the last two millennia (e.g., Ljungqvist^1^) are important, as such events directly affect human life. As an area strongly influenced by the East Asian monsoon system, northeast Asian is characterized by considerable precipitation variability, which leads to frequent dry and wet periods and severe droughts and floods that have major socioeconomic impacts due to their effects on agriculture, etc. The position of the rainband of the East Asian summer monsoon (EASM) is critical to the rapid hydroclimatic variability, which has important effects on society; for example, the collapses of historical Korean and Chinese dynasties was linked to extreme climate events^2–8^.

As the Korean Peninsula (KP), which is located on the East Eurasian continental margin facing the Western Pacific Ocean, is simultaneously affected by both terrestrial and oceanic environmental factors, it has a history of more dynamic climate oscillations than China and Japan^9–14^. Therefore, the KP is an ideal site for studies of East Asian monsoon system activity in East Asia. Due to high and continuous sedimentation and climatic sensitivity, lake (reservoir) sediments and speleothems provide a record of multi decadal- to centennial-scale East Asian monsoon system changes. However, few investigations of lake sediments have dealt with geoecological environmental responses to rapid climatic changes during the mid- to late Holocene in the KP^15–18^.

We obtained the GG-19–2-1 sediment core from the Gonggeom-ji paleo-reservoir, which was embanked during the Unified Silla dynasty (ca. 700 CE) for flood control and to provide irrigation for rice cultivation^19^. Age-controlled lithological analysis assumed that the paleo-reservoir was initially formed as a fluvial floodplain at around 2230 cal yr BP, subsequently changing to a reservoir with a high sedimentation rate (2–8 mm/yr) at about 1500 cal yr BP.

Pollen analysis was carried out to estimate pollen-based quantitative annual precipitation (PqPann) over the last 1300 years (650–1940 CE). However, we found that the GG19-2–1 core has a hiatus, with records missing for the 660 years from 980 to 1650 CE. Until now, if a hiatus was present in a sediment succession, it was impossible to reconstruct the paleoenvironment for the corresponding period. To overcome the lack of information, for the first time, we used a machine-learning method (artificial neural networks, ANNs), and a well-established and age-controlled high-resolution pollen dataset, to reconstruct the quantitative annual precipitation (Pann) during the period 980–1650 CE. Changes in precipitation on the KP over the past 1300 years were reconstructed, including through pollen analysis and covering the hiatus period. When the same statistical technique was applied to the reconstructed pollen-based precipitation and δ^18^O of speleothem data, we found time-series lags between biotic and abiotic responses to rainfall, although they showed similar fluctuation trends. The time-series lags differed between the Medieval Warm Period (MWP) and Little Ice Age (LIA). As no studies on this topic have been published, we discuss the new findings from a geoecological viewpoint. Finally, the reconstructed precipitation data suggested that the magnitude of rainfall differed depending on the location of the EASM rainband over the past 1300 years in Northeast Asia.

## Results

In general, the pollen assemblage over the past 1300 years (Supplementary Figure S2) reflects a mixed forest of conifers and broadleaf deciduous trees dominated by pine (*Pinus*), oak (*Quercus*), and apricot (*Prunus*) in a mountainous area with an understory of ferns. In addition, herbs, including grasses (Poaceae) and sedges (Cyperaceae) associated with riparian willow (*Salix*), alder (*Alnus*), and smartweed (*Persicaria*), grew in lowland areas and along streams. Mountainous trees and shrubs were predominant over the last 1300 years, although the relative proportions of dominant taxa changed over time. The concentration (pollen grains per unit of sediment volume) of the palynoflora increased suddenly at a hiatus depth of 3.5 m, which is the boundary of the local pollen assemblage zone of GG19-III and GG19-IV.

**Figure 1:**
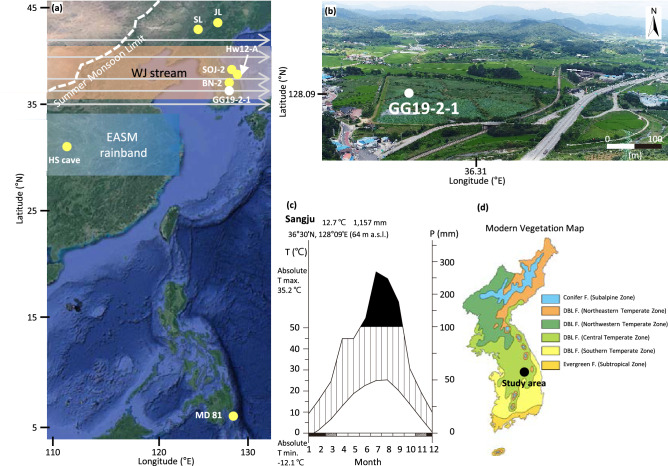
(**a**) Locations of the GG19-2–1 core site and other sites cited in the text: Hw12-A (Hwajinpo) and SOJ-2 (Songji) lakes, and BN-2 (Baeg-nyeong) cave, South Korea; SL (Silhailongwan) and JL (Jingpo) lakes, northeast China, and HE (Heshang) cave, central China; and MD81 in the western tropical Pacific. These maps were created using Generic Mapping Tools (v.5.4.4, https://www.generic-mapping-tools.org/download/). The dashed line indicates the northernmost extent of the Asian summer monsoon. Position and orientation of the rainband and westerly jet (WJ) stream during the Late Holocene are from Herzschuh et al.^31^ Arrowed grey parallel lines represent the general direction and width of the WJ stream. (**b**) Aerial photograph of Gonggeom-ji showing the location of the GG19-2–1 core site. (**c**) Seasonal changes (2002–2019 CE) in temperature and precipitation in the Sangju area, where the Gonggeom-ji paleo-reservoir is located, based on information from the Korea Meteorological Administration (https://data.kma.go.kr). (**d**) The study area belongs to the central temperate zone of the deciduous broadleaf forest based on the modern vegetation map of Korea (modified from Yim and Kira^69^) (drawn using CorelDRAW Graphics Suite X7). DBL F., deciduous broadleaved forest.

**Figure 2:**
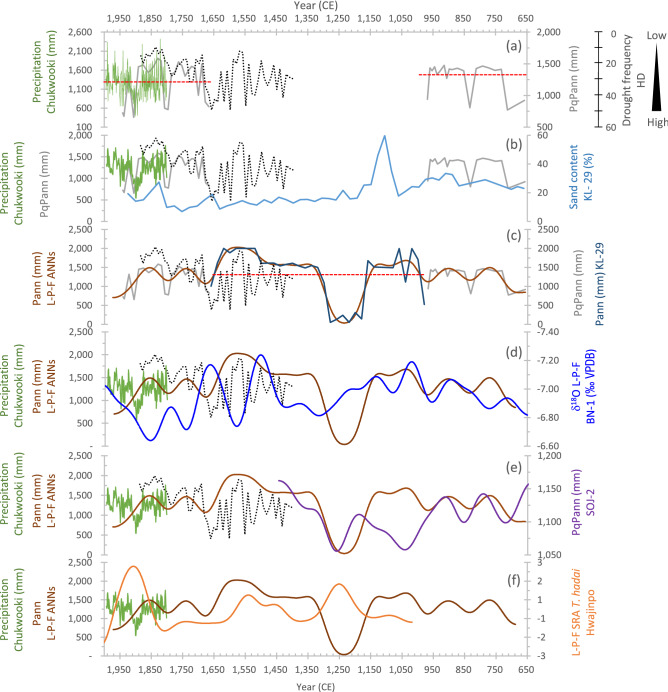
Pollen-based quantitative annual precipitation (PqPann) and artificial neural networks (ANNs) Pann reconstruction from 650 to 1950 CE and their verification by comparison with the Korean proxy results of the relative sand contents (%) of KL-29^22^, oxygen isotopes (δ^18^O ‰ VPDB) of Baeg-nyeong Cave (BN-1)^9^, the foraminifera index^16^, old (Chukwooki; 1777–1910) and modern (1908–1995) rain gauge data^20,21^, and drought records from the annals of the Joseon Dynasty^8^. (**a**) PqPann (light gray line), old rain gauge data (green line), and frequency of droughts (black dotted line). The red dotted line indicates the average PqPann. (**b**) PqPann (mm) and sand contents (%) of KL-29. (**c**) PqPann (light gray line), Pann of KL-29 (dark blue line), and 100-year low-pass-filtered (L-P-F) data (dark brown line) of ANNs. The red dotted line indicates the average ANNs. (**d**) L-P-F at 100 years ANNs precipitation (mm) of the GG19-2–1 sediment core (dark brown line) and oxygen isotope (δ^18^O ‰ VPDB) of BN-1 (blue line) showing a lag time of ca. 25–100 years. (**e**) L-P-F at 100 years PqPann comparison between the northeast coast, Songji Lake^18^, and inland (GG19-2–1, this study) region of the KP. (**f**) L-P-F at 100 years ANNs and the foraminifera *Trochammina hadai* from Hwajinpo Lake (drawn using CorelDRAW Graphics Suite X7).

Comparison of Pann and independent data (calculated with 100-year Butterworth low-pass filtering to match the time resolution of dry–wet periods), shown in Figs. 1, 2, indicates that the dry–wet periods could provide a reasonable representation of precipitation change for the calibrated period. The lag times between Pann and the oxygen isotope data were 17.26 years during the MWP and 94.06 years during the LIA (Supplementary Figure S3b, c).

**Figure 3:**
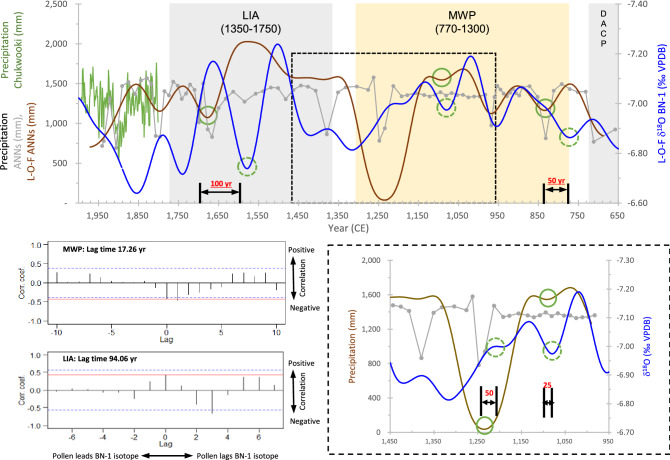
Comparison of 100-year L-P-F values between the ANNs (mm) and BN-1 (δ^18^O ‰ VPDB) during the period 650–1950 CE. Peaks of BN-1 are about 25–100 years ahead of those of PqPann. Cross-correlation results show that lag times of LIA (94.06 yr) are longer than those of MWP (17.26 yr). The red lines represent the correlation coefficient for lag 0. Correlation coefficients outside the dashed blue lines are significant at *P* = 0.05. Amplitudes from Pann of ANNs and oxygen isotope of BN-1 during the LIA are generally higher than during the MWP in the KP. LIA, Little Ice Age; MWP, Medieval Warm Period; DACP, Dark Age Cold Period (drawn using CorelDRAW Graphics Suite X7).

PqPann reconstruction was performed for the periods 1940–1666 and 968–658 CE, which are separated by the hiatus (Supplementary Figure S1). The average PqPann was 1,222 mm/yr (range: 655–1,530 mm/yr) during the period 1940–1666 CE, and 1304 mm/yr (range: 770–1,454 mm/yr) during the period 968–658 CE (Fig. 2a, b). The ANNs was used to estimate the Pann for the hiatus period 1650–983 CE, for which pollen data are not available (Fig. 2c). The reconstructed Pann averaged 1,290 mm/yr (range: 670–1,580 mm/yr). The PqPann- and ANNs-reconstructed datasets had similar amplitudes, ranging from 655 to 1,580 mm/yr. PqPann data were verified by comparison with old (Chukwooki; 1777–1910 CE) and modern (1908–1995 CE) rain gauge data^20,21^, and with the drought frequency in the annals of the Joseon Dynasty (1400–1890 CE)^8^ (Fig. 2a). The relative sand contents (%) of KL-29^22^ were used as input variables to estimate the ANNs data and we found good matches (Fig. 2b). Subsequently, a 100-year Butterworth low-pass filter was applied for PqPann (Fig. 2c) and was correlated with that of the δ^18^O (‰) of BN-1 of Baengnyeong Cave^9^, revealing biotic lag ranging from 25 to 100 years (Figs. 2d, 3, and Supplementary Figure S3). With Butterworth low-pass filtering, our data were comparable to those for Lakes Songji^18^ and Hwajinpo^16^, with the exception of a few periods (e.g., 1150–990 CE in Fig. 2e and 1290–1190 CE in Fig. 2f).

## Discussion and conclusions

### PqPann and ANNs reconstruction and influencing factors

Over the last decade, PqPann reconstruction has been performed for East Asia^23–31^. The reconstructed dataset suggests that centennial- to millennial-scale dry–wet fluctuations are linked to the variability in the EASM and north–southward migration of the westerly jet stream (WJ) that was teleconnected to the Atlantic Meridional Overturning Circulation during the Holocene.

**Figure 4:**
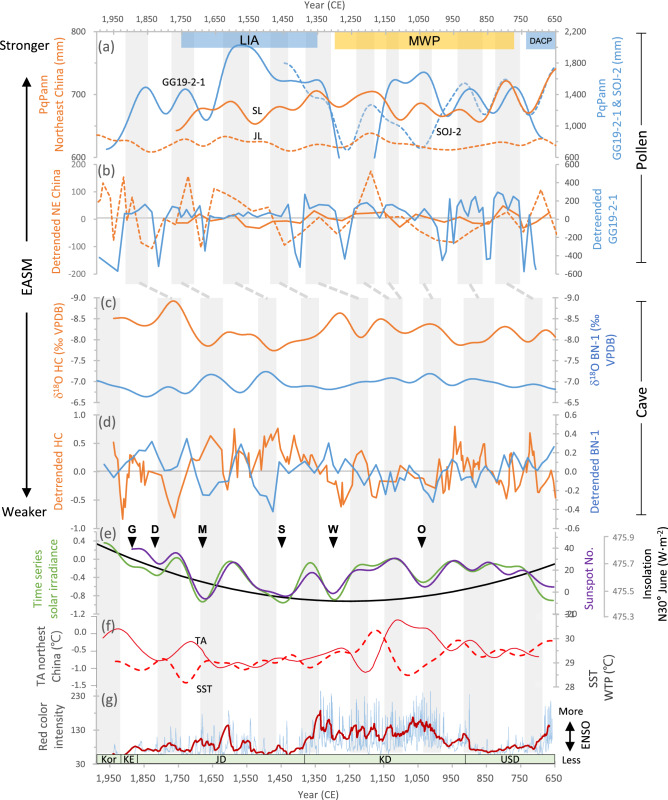
Multi-centennial dry–wet periods and time-series lags of biotic (pollen) and abiotic (speleothem) responses to rainfall over the past 1300 years in East Asia. (**a**) PqPann of Korean^18^ (GG19-2–1 sediment core, solid blue line) and Chinese^27,32^ pollen data. SL (solid brown line), Sihailongwan Lake. JL (dashed brown line), Jingpo Lake; SOJ-2 (dashed blue line), Songji Lake. (**b**) Detrended fluctuations of PqPann showing the frequency, amplitude, and duration of Pann at four sites. All colors and lines have the same meaning as in Fig. 4 (**a**). (**c**) δ^18^O of the Korean Baeg-nyeong Cave^9^ and Chinese Heshang Cave^33^. A low δ^18^O corresponds to a strong EASM. (**d**) Detrended fluctuations of the two caves showing the amplitude and duration of the high precipitation interval. (**e**) Time series of solar irradiance^70^, number of sunspots^50^, and insolation at N30° in June^35^ showing synchronous fluctuations. Upper case letters indicate grand solar minima: O, Oort; W, Wolf; S, Spörer; M, Maunder; D, Dalton; G, Gleissberg. (**f**) Temperature anomaly (℃) of northeast China^7^ and sea surface temperature (℃) of the western tropical Pacific^71^. (**g**) ENSO intensity from the Southern Hemisphere^72^ with 100-point moving average values (thick purple line). Light-gray bars indicate the high-precipitation periods represented by the Korean PqPann (GG19-2–1) and speleothem (BN-1) data. EASM, East Asian Summer monsoon; ENSO, El Niño-Southern Oscillation. The Korean chronology is plotted at the bottom. Kor, Korea; KE, Korea Empire; JD, Joseon Dynasty; KD, Koryŏ Dynasty; and USD, Unified Silla Dynasty (drawn using CorelDRAW Graphics Suite X7).

As an area strongly influenced by the East Asian monsoon system, the KP has characteristically high precipitation variability, leading to higher frequencies and amplitudes of dry and wet periods^20^, and severe, persistent droughts and floods, described in historical documents^8^ (Fig. 2). PqPann and ANNs reconstruction for the period 650–1940 CE in South Korea included 10 centennial-scale wet epochs (light gray bars in Fig. 4a), with multi-decadal dry/wet fluctuations within each epoch (Fig. 4b). The variability and trend of the PqPann and ANNs reconstruction of South Korea (GG19-2–1 and SOJ-2^18^) are comparable to those of northeast China (Lakes Jingpo^32^ and Silhailongwan^27^), but the Korean PqPann amplitudes (655–1530 mm/yr) are higher than those in China (644–949 mm/yr) (Fig. 4a), despite the almost equivalent rainfall duration (Fig. 4b).

The discrepancy in rainfall intensity between the two regions may be due to the difference in distance from the EASM rainband. According to Herzschuh et al.^31^, the EASM rainband that influenced the intensity and amount of precipitation during the late Holocene was located in southeastern China (Fig. 1). Therefore, the KP is much closer to the EASM rainband than is northeast China, so the KP is highly affected by rainfall forcing of the EASM rainband. In comparison with BN-1^9^ of South Korea, δ^18^O of HC^33^ for southeastern China had small values, but a higher amplitude (Fig. 4c, d), indicating greater rainfall intensity in southeastern China due to its location within the EASM rainband area (Fig. 1).

Gradual weakening of EASM intensity was detected from the mid to late Holocene^34^, corresponding to a decrease in Northern Hemisphere summer insolation^35^ and enhanced El Niño–Southern Oscillation (ENSO) activity^14,36–38^. However, the solar forcing^29^ and ENSO-driven hydroclimatic variability^18,39^ during the late Holocene increased the precipitation intensity over the KP, which is located on the East Asian coast. The atmospheric (continental setting) and oceanic (East Asian coast) forcings influencing the multi-centennial dry and wet epochs are recorded in GG19-2–1 (Figs. 4e,f,g).

### Biotic lags in response to rapid climate changes

The biotic responses to rapid climate change on a geological timescale around the North Atlantic have been investigated at an intermediate temporal scale^40–46^. Williams et al.^40^ reported that vegetation changes took place < 100 years after the climatic oscillation, together with major climatic changes. Based on pollen and plant macrofossil data plus oxygen isotope data, Yu^41^ also provided evidence for replacement of spruce forest by pine within ~ 100 years of the beginning of the Holocene warming. These studies documented a response of the plant population to climate change on an intermediate temporal scale that was more gradual than the immediate individual (phenology) response to recent warming^47^. Meanwhile, Post^40^ argued that the responses of plant communities to climate change are as fast as, or even faster than, the responses predicted by the stochastic forest gap models of Lischke et al.^48^, and that the plant population shifts in response to climatic oscillation on the order of decades are equally important in biomes.

The time series of the PqPann GG19-2–1 and δ^18^O data for BN-1^9^ were compared using 100-year Butterworth low-pass filtering and cross-correlation analysis. Remarkably, the medians of the most highly significant cross-correlations between the two datasets revealed similar variability, with lags of 25–50 and 50–100 years during the MWP and LIA, respectively (Fig. 3).

In general, the PqPann fluctuations matched temperature anomaly fluctuations in northeast China^7^ (Fig. 4a, f), whereas the time-series variability data of δ^18^O for BN-1 was comparable to the time series solar irradiance^49^ and sunspot number^50^ data (Fig. 4e), resulting in BN-1 leads with lags in the variation of PqPann. The 10 peaks of PqPann (Fig. 4a) always followed those of BN-1 (Fig. 4c) during the period 650–1940 CE in South Korea, because shifts in plant community responses to abrupt climate changes occurred during multi-decadal dry and wet epochs^40,44^. The response times of plant communities are also dependent on temperature, with warmer and colder periods associated with faster and slower responses, respectively.

We only used pollen data derived from trees and shrubs (arboreal pollen, AP) to reconstruct precipitation, because trees and shrubs are sensitive to climate change, whereas grasses (non-arboreal pollen, NAP) are generally climate-tolerant. In Korea, mountain areas have been used for slash-and-burn agriculture to secure fields for cultivation^51,52^. In mountain areas, the forests are mainly trees and shrubs, and are influenced by human interference, changes in the spectrum of AP, and enhanced surface soil erosion, which all increase the sedimentation rate^53^. By contrast, in the study area, riverine herbs and aquatic and cultivated grasses occupied the lowlands and paleo-reservoir (paleo-wetland). In addition, at the time of the analysis, the study area was mainly used for rice paddy farming^54^. Therefore, we inferred that the trees and shrubs used to reconstruct precipitation were relatively unaffected by human activities; therefore, the trees and shrubs likely responded to changes in the natural environment. Ammann et al.^55^ reported that the individual responses of plant species to climatic changes may reflect processes in individuals (e.g., productivity and phenology) and populations (e.g., population dynamics), as well as spatial distributions (e.g., migrations). Our pollen data suggested effects of the first two processes only, as the study area has the geomorphological characteristics of a small basin surrounded by hills and low mountains.

Considering that speleothem δ^18^O records rapidly reflect water-controlled abiotic processes in the atmosphere (vadose zone), they can signal hydroclimatic changes with much shorter response times relative to those of vegetation. A case study of the modern speleothem geochemistry near our study area implies maximum response times of a few months to rainfall events during rainy summers^56^. Therefore, the discovery of lags between biotic (PqPann data of GG19-2–1) and abiotic (δ^18^O data of BN-1) responses in this study are strongly supported by the results of Williams et al.^40^, who reported a vegetation response time of 60 years in the most highly significant cross-correlation among lake sediments and a median lag time of 90 years among all significant cross-correlations between pollen and δ^18^O and chironomid assemblages.

In an ecological context, changes on a multi-decadal time scale in response to climate oscillations represent no more than a few population cycles for many arboreal taxa^40^. However, we suggest that the reconstruction of decadal- to centennial-scale climatic oscillations should be taken into account in determining biotic lags, even though they can be ignored in the reconstruction of millennial-scale climatic variability. Our new finding can be used to predict shifts in vegetation responses to climatic changes such as rapid warming or cooling under conditions of global warming.

## Methods

The Gonggeom-ji paleo-reservoir is located inland in central South Korea (36°30′46"N, 128°09′39"E, elevation 64 m above sea level) (Fig. 1). The 7-m-long GG19-2–1 sediment core obtained from Gonggeom-ji consists mainly of medium to coarse sand in the lower section, gray to dark-gray laminated clayey silt in the middle section, and oxidized brown mottled and laminated clayey silt in the upper section (Supplementary Figure S1).

The GG19-2–1 sediment core was dated by accelerator mass spectrometry (AMS) analysis of ^14^C in eight plant samples held by the Korea Institute of Geoscience and Mineral Resources, and calendric ages were recalculated using CalPal-2007Online^57^ (Supplementary Table S1). The ^137^Cs activity was measured in eight soil samples taken at 2.5-cm intervals from the upper Sect. (1.1–1.3 m) of the core, excluding the disturbed soil layer (Supplementary Figure S1). The depth at which ^137^Cs activity was first detected, 1.225 m, was assigned to the year 1963. An age–depth model was constructed using the R package Clam^58^ and IntCal13 radiocarbon calibration curve^59^ analysis, i.e., AMS analysis of the ^14^C and ^137^Cs activity of eight samples.

Fifty-five samples of the GG19-2–1 sediment core were taken for pollen analysis at 10-cm intervals from 1.0 to 6.5 m. Pollen was extracted using a standard palynological method^60^. Two tablets containing exotic *Lycopodium* spores (27,637 per tablet) were added to each dry sample (2–4 g) to calculate the palynological concentration per gram of sample^61^. A pollen diagram with local pollen assemblage zones was produced using Tilia software^62^ (Supplementary Figure S2).

Annual precipitation over the last 1300 years was quantitatively reconstructed based on the subfossil AP records using CREST software^63^ and the CREST-formatted GBIF database^64^. The dataset contains the records of plants with quarter-degree grid cell resolution; we used 5,123 plant distribution records within 450 grid cells, which correspond to the geographical range of the KP. To determine the rationality of the PqPann reconstruction, we compared the instrumental rainfall and historic drought records (Fig. 2a–c) and reconstructed the precipitation index in the central inland and northeast coast areas from sediment, cave, and microfossil proxy data (Fig. 2d–f). The sand contents of cores KL-28 and KL-29^22,65^, which were used as indicators of the Holocene precipitation intensity, were chosen as input variables (Fig. 2b). Both of these cores, and the GG19-2–1 core, are located at the same latitude in the central inland region of the KP, and appear to have experienced similar climate changes during the late Holocene. The sand contents of the two cores were interpolated linearly according to the age of the pollen data.

The age-depth model^58^, together with the considered core section showing an uneven irregular erosional surface and dramatic shifts of grain size (μm) and mean Folk and Ward (Φ), indicated a 660-year hiatus at a depth of 3.5 m in the core sample (Supplementary Figure S1). An ANNs was used to reconstruct Pann for the hiatus section of the GG19-2–1 sediment core using Visual Gene Developer 1.9 build 785^66^ (Fig. 2c). The structure of the ANNs was designed with two hidden layers. PqPann and ANNs precipitation data were highly correlated with each other (R^2^ = 0.955) (Supplementary Figure S3a). The sand contents of core KL-29^22^, which were used as indicators of the Holocene precipitation intensity, were chosen as the input variables (Fig. 2b). The neural network was trained with a back-propagation algorithm using a hyperbolic tangent transfer function.

We used a Butterworth low-pass-filter to determine the periodicity of precipitation variability and performed cross-correlation analysis to identify the lag time between biotic and abiotic responses to precipitation. We performed power spectral analysis of the ANNs and BN-1 δ^18^O data to determine the optimal Butterworth low-pass-filter value. Power spectral analysis of the ANNs and BN-1 δ^18^O data for the last 1300 years (650–1940 CE) yielded periodicities of ca. 90–150 years, ranging from 93 to 144 years and from 117 to 144 years (above the 95% confidence level), respectively (Supplementary Figure S4). Based on these results, a 100-year Butterworth low-pass filter was applied (Fig. 2d,e,f) using the R package signal^67^. We also performed cross-correlation analysis (Fig. 3 and S3) using BINCOR^68^, to determine the lag time between the biotic and abiotic responses to precipitation.

## Supplementary information

Supplementary Information
