# Supplementary material for: Asynchronous multi-decadal time-scale series of biotic and abiotic responses to precipitation during the last 1300 years
Source: Sci Rep. 2020 Oct 20;10:17814. doi: 10.1038/s41598-020-74994-x (PMC7576146; doi:10.1038/s41598-020-74994-x)
Supplement: Supplementary file 1 — Supplementary Information [file 41598_2020_74994_MOESM1_ESM.doc]

Supplementary Information for

**Asynchronous Multi-decadal Time-scale Series of Biotic and Abiotic Responses to Precipitation during the last 1300 Years**

## Sangheon Yi^1, 2^, Chang-Pyo Jun^1^, Kyoung-nam Jo^3^, Hoil Lee^1^, Min-Seok Kim^1^, Sang Deuk Lee^4^, Xianyong Cao^5, 6^, and Jaesoo Lim^1^

## ^1^Korea Institute of Geoscience and Mineral Resources, Daejeon 34132, Korea

## ^2^Korea University of Science and Technology (UST), Daejeon 34113, Korea

## ^3^Kangwon National University, Chuncheon 24341, Korea

## ^4^Nakdonggang National Institute of Biological Resources, Sangju 37242, Korea

## ^5^Key Laboratory of Alpine Ecology, Institute of Tibetan Plateau Research, Beijing, China

## ^6^CAS Center for Excellence in Tibetan Plateau Earth Sciences, Beijing 100101, China

*^*^*Correspondence to shyi@kigam.re.kr, cpjun@kigam.re.kr

**Supplementary figures**

1. **Figure S1.** Lithology column, calibrated age, age–depth model (Clam software), core section, median grain size (μm), mean Folk and Ward (Φ), and lithological characteristics.
2. **Figure S2.** Selected pollen diagram of GG19-2-1 sediment core from Gonggeom-Ji
3. **Figure S3.** Strong correlation of precipitation reconstruction between PqPann and ANN (R^2^ = 0.955)
4. **Figure S4.** Spectrum analysis of the oxygen isotope periodicity in Baeg-nyong cave (BN-1) and ANNs PqPann of GG19-2-1 sediment core (this study)

**Supplementary table**

1. **Table S1.** AMS radiocarbon dates of GG19-02-1 core from the paleoreservoir Gonggeom-ji

**Supplementary reference list**

**Figure S1.** Lithology column, calibrated age, age–depth model (Clam software), core section, median grain size (μm), mean Folk and Ward (Φ), and lithological characteristics. C, clay; Z, silt; fS, fine sand; cS, coarse sand. A hiatus at a depth of 3.5 m was recognized by radiocarbon dates, together with an uneven irregular erosional surface (ES), and obvious peaks of median grain size and mean Φ.


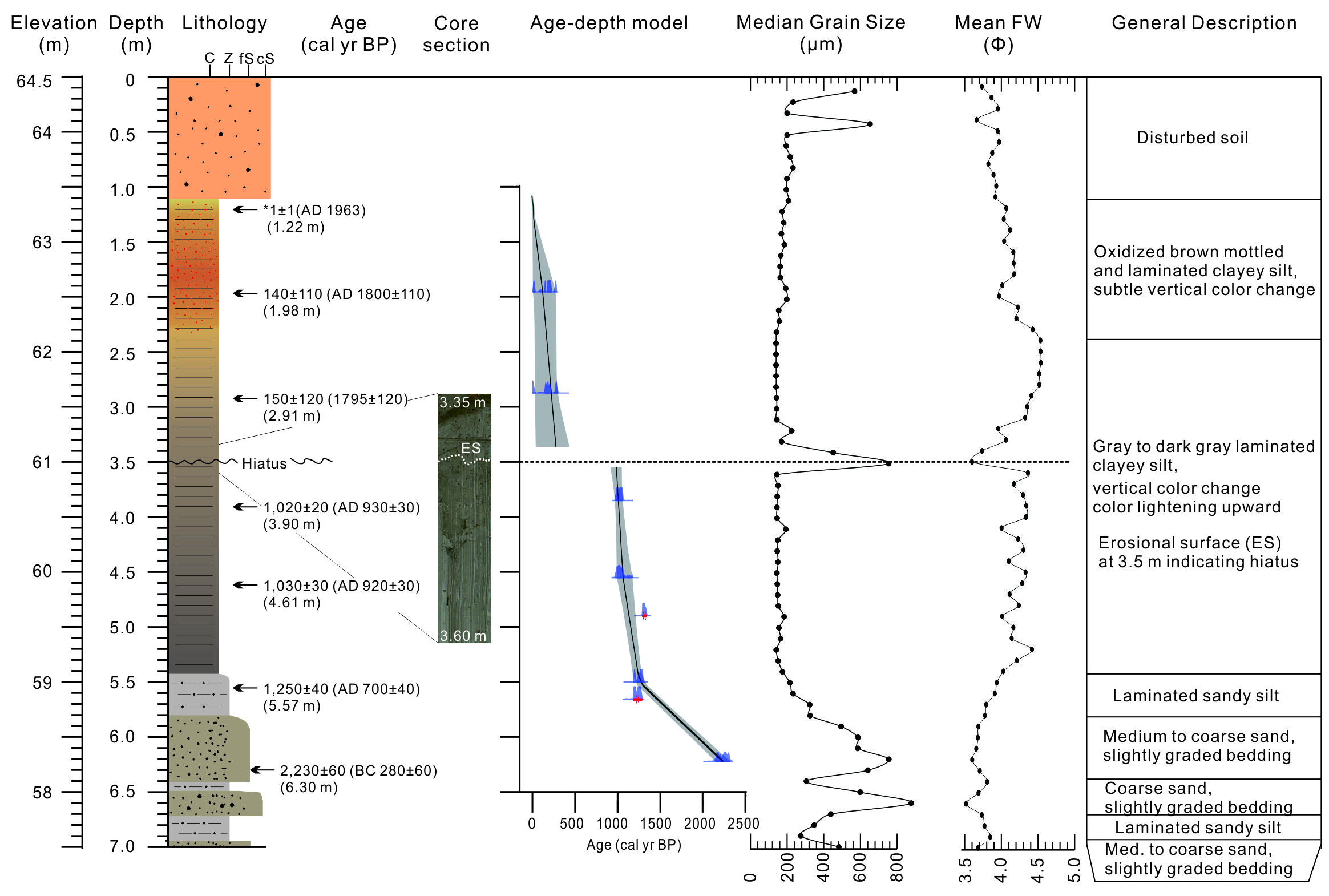


**Figure S2.** Selected pollen diagram of GG19-2-1 sediment core from Gonggeom-Ji. Only major and critical taxa are shown. Calibrated radiocarbon dates are shown on the left-hand side of the diagram. The dendrogram on the right-hand side was generated by constrained cluster analysis with CONISS in Tilia^1^. Asterisks indicate the ^137^Cs age. T-C-C, Taxaceae-Cephalotaxaceae-Cupressaceae. LPAZ, local pollen assemblage zone.

**Figure S3.** (a) Strong correlation of precipitation reconstruction between PqPann and ANNs (R2 = 0.955). (b) and (c) Cross-correlation analyses of the ANNs and BN-1 during the periods of MWP and LIA, showing lag times of 17.26 yr and 94.06 yr, respectively. The red lines represent the correlation coefficient for lag 0. Correlation coefficients outside the dashed blue lines are significant at *P = 0.05*.

***
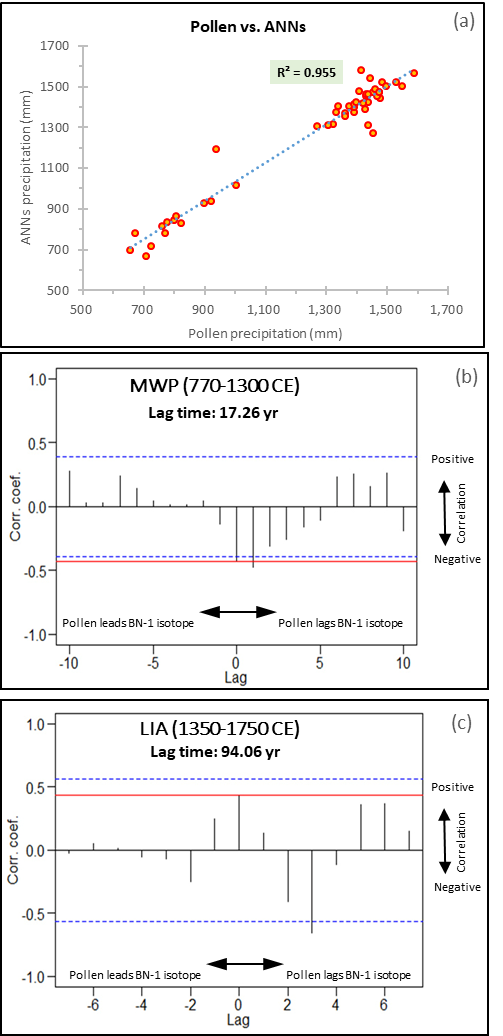
***

**Figure S4.** Spectrum analysis of the oxygen isotope periodicity in Baeg-nyeong Cave (BN-1)^2^ and ANNs of the GG19-2-1 sediment core based on PqPann (this study). The two proxies show a periodicity of about 90–150 years with 95% confidence level.

***
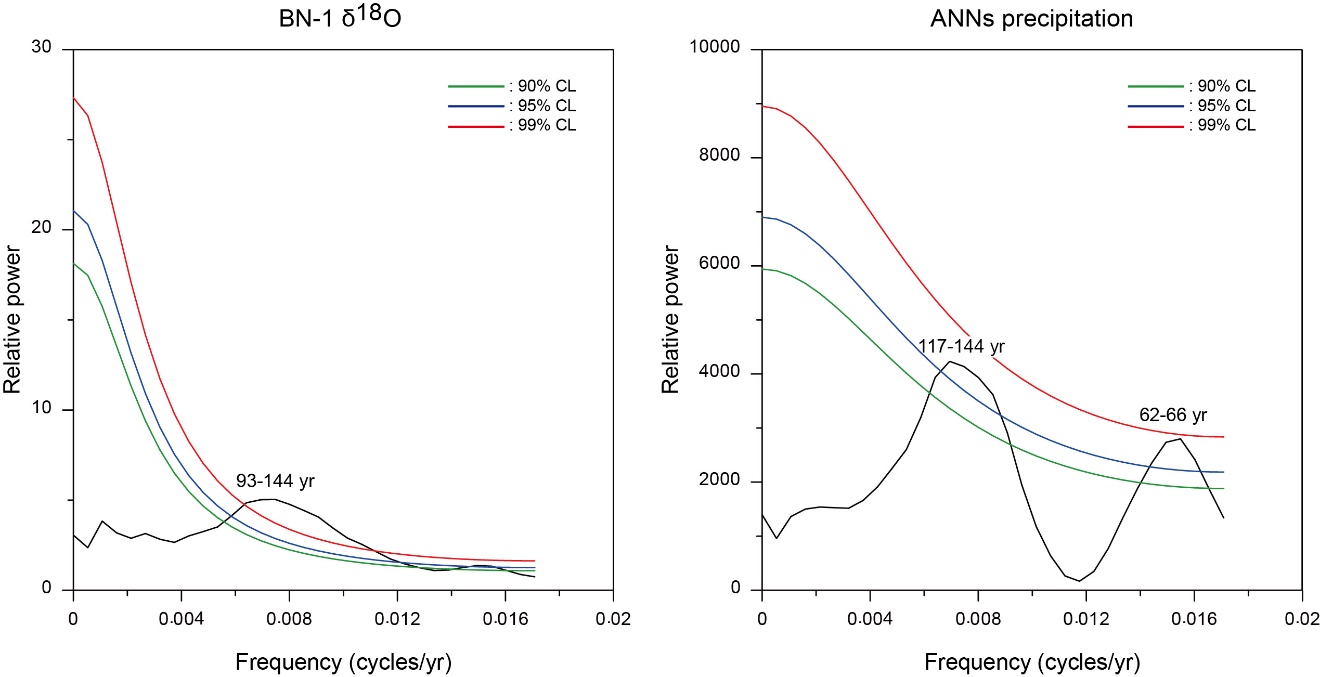
***

**Table S1** AMS radiocarbon dates of GG19-02-1 core from the paleoreservoir Gonggeom-ji. Calendric ages are calculated by CalPal-2007online^3^.

| Elevation | Depth | 14C yr BP | Cal. yr BP | Calendric age | Laboratory | Material |
| --- | --- | --- | --- | --- | --- | --- |
| (m) | (m) | (±1σ) | (±2σ) | (AD/BC) | code |  |
| 63.22 | 1.22 |  |  | *AD 1963 | GG19-02-1_122d5 | Soil |
| 62.40 | 1.98 | 160±20 | 140±110 | AD 1800±110 | KGM-OWd190304 | Plant fragment |
| 61.47 | 2.91 | 190±30 | 150±120 | AD 1795±120 | KGM-OWd190305 | Plant fragment |
| 60.48 | 3.90 | 1,120±20 | 1,020±20 | AD 930±30 | KGM-OWd190306 | Plant fragment |
| 60.09 | 4.61 | 1,130±30 | 1,030±30 | AD 920±30 | KGM-OWd190308 | Plant fragment |
| 59.77 | 4.96 | 1,390±20 | 1,310±10 | AD 640±10 | KGM-OWd190309 | Plant fragment |
| 59.42 | 5.57 | 1,320±30 | 1,250±40 | AD 700±40 | KGM-OWd190310 | Plant fragment |
| 58.81 | 5.73 | 1,280±30 | 1,230±30 | AD 720±30 | KGM-OWd190311 | Plant fragment |
| 58.65 | 6.30 | 2,200±30 | 2,230±60 | BC 280±60 | KGM-OWd190312 | Plant fragment |
| *Cesium 137 dating | |  |  |  |  |  |

**References**

1. Grimm, E. Tilia 1.7.16 Software. Illinois State Museum, Research and Collection Center, Springfield, II. (2011).

2. Jo, K. *et al.* 1000-Year Quasi-Periodicity of Weak Monsoon Events in Temperate Northeast Asia since the Mid-Holocene. *Sci. Rep.* **7**, 15196 (2017).

3. Danzeglocke, U., Joris, O. & Weninger, B. CalPal-2007online. Available at: http://www.calpal-online.de.
